# Peoria County Medical Society

**Published:** 1867-09

**Authors:** G. L. Corcoran, H. Steele

**Affiliations:** Chairman; Secretary


### PEORIA COUNTY MEDICAL SOCIETY.

Pursuant to call, the following medical gentlemen assembled at the office of Dr. H. Steele, in Elmwood, Peoria County, Ill., June 15th, at 1 o'clock P.M., for tne purpose of organizing a Medical Society:

Dr. G. L. Corcoran, Brimfield; Dr. F. A. Warner, Farmington; Dr. John Gillette, Tivoli; Drs. Hensley and Hoit, Yates City; Dr. J. K. Secord, Elmwood; Dr. H. Steele, Elmwood.

On motion, Dr. Corcoran was chosen temporary Chairman, and Dr. Steele, Secretary.

After the adoption of a Constitution and By-Laws, the Society proceeded to the election of permanent officers for the ensuing year, which resulted in the selection of the following gentlemen:

*President*—G. L. Corcoran.
*Vice President*—John Gillette.
*Secretary*—F. A. Warner.
*Treasurer*—H. Steel.
*Censors*—W. Husley, Hoit, and J. K. Secord.

Communications were received from several medical gentlemen, who had been unable to be present, to the effect that they would endorse the proceedings of the Society.

The Society then proceeded to the adoption of a fee bill.

The following subjects were selected for discussion at the next regular meeting:

Administration of Medicine by the Hypodermic Method.
Bromide Potassium.
Cerebro-Spinal Meningitis.

After a brief discussion upon various medical topics, it was ordered that a synopsis of the proceedings be furnished the Chicago MEDICAL JOURNAL and Yates City *Herald*, for publication.

On motion, the Society adjourned, to meet at Brimfield, on Wednesday, Sept. 4th, 1867, at 1 o'clock P.M.

G. L. CORCORAN, Chairman.

H. STEELE, Secretary.

